# Application of the Ridden Horse Pain Ethogram to Elite Dressage Horses Competing in World Cup Grand Prix Competitions

**DOI:** 10.3390/ani11051187

**Published:** 2021-04-21

**Authors:** Sue Dyson, Danica Pollard

**Affiliations:** 1 The Cottage, Church Road, Market Weston, Diss IP22 2NX, UK; 2 The Rodhams, Rodham Road, Christchurch, Wisbech PE14 9NU, UK; drdee.pollard@gmail.com

**Keywords:** Equine, lameness, behaviour, head position, walk, trot, canter, flying changes, piaffe, passage

## Abstract

**Simple Summary:**

A Ridden Horse Pain Ethogram (RHpE) was developed comprising 24 behaviours; a score of ≥8/24 is likely to reflect the presence of musculoskeletal pain. The aim of the study was to apply the RHpE to elite dressage horses, competing at World Cup Grand Prix qualifying competitions or finals. It was hypothesised that this should be a group of horses with a low incidence of musculoskeletal pain; thus, RHpE scores would be consistently <8. Additional aims were to compare RHpE scores with judges’ scores and to compare these and other observations concerning gait with the guidelines for judging Fédération Equestre Internationale (FEI) dressage. The RHpE was applied by a trained assessor to video recordings of 147 competitors at nine venues. Freehand notes described additional observations. The median RHpE score for all competitors was 3/24 (range 0, 7). There was a moderate negative correlation between the RHpE score and the judges’ score. There was a high frequency of occurrence of head behind vertical ≥10° ≥10 s, mouth open with separation of the teeth ≥10 s and repeated tail swishing, behaviours that should be penalised according to FEI rules. It was nonetheless concluded that most horses appeared to work comfortably for the majority of the test.

**Abstract:**

There is considerable debate about the social license to compete with horses and controversy about training methods for dressage horses. The objectives were to: 1. apply the Ridden Horse Pain Ethogram (RHpE) to dressage horses competing at elite Grand Prix level; 2. compare RHpE and judges’ scores; and 3. document deviations in gaits from Fédération Equestre Internationale (FEI) guidelines. Video recordings of 147 competitors from nine World Cup competitions were assessed. Spearman’s rank correlation coefficient tested the correlation between RHpE and judges’ scores. The median RHpE score was 3 (IQR 1, 4; range 0, 7). There was a moderate negative correlation (Spearman rho −0.40, *p* < 0.001) between the RHpE scores and the judges’ scores. Mouth open with separation of the teeth for ≥10 s (68%), head behind vertical ≥10° ≥10 s (67%), an intense stare for ≥5 s (30%) and repeated tail swishing (29%) were the most frequent RHpE behaviours. Deviations from FEI guidelines were most frequent in passage, piaffe, canter flying-changes, canter pirouettes and “halt-immobility-rein back five steps-collected trot”. In conclusion, most horses appeared to work comfortably for the majority of the test. Further investigation of the influence of a double bridle compared with a snaffle bridle on head position and mouth opening is merited.

## 1. Introduction

A Ridden Horse Pain Ethogram (RHpE) comprising 24 behaviours, the majority of which are at least 10 times more likely to be seen in lame versus non-lame horses, has previously been developed [1]. It has been shown that a RHpE score ≥8 is likely to reflect the presence of musculoskeletal pain [1,2,3,4,5,6]. Removal of pain by diagnostic anaesthesia results in significant reductions in RHpE scores [2,3], indicating a causal relationship between behaviour and pain. Rider skill did not alter RHpE scores, although different behaviours may be manifest when a horse is ridden by two riders of varying skill [7].

In a previous live pilot study assessing horses (*n* = 35) warming-up for dressage at a 4* (now 5*) three-day event in 2018, horses that had a RHpE score ≥7 were more likely to be eliminated or retire in the cross-country phase than horses with a RHpE score <7 [8]. The RHpE was subsequently applied to all horses (*n* = 137) competing at two 5* three-day events in 2019 [9]. There was a significant association between the RHpE score during warm-up for dressage and dressage penalties and final placing. Horses with a RHpE score of ≥7 were more likely to be eliminated or retire during cross-country than horses scoring <7. There was also an association between lameness or gait abnormalities in canter and a RHpE score ≥ 7. There was good consistency of results for horses which competed at both events. It was, therefore, concluded that the use of the RHpE may help to identify elite horses which might benefit from investigation and treatment in order to both improve performance and enhance equine welfare.

In contrast, in a study of a convenience sample of 148 sports and leisure horses in the UK, believed by their owners (both amateur and professional riders) to be working comfortably, 62% were lame when ridden, and 60% of horses showed gait abnormalities in canter [5]. The median RHpE score was 8/24 (interquartile range [IQR] 5, 9; range 0, 15). There was a positive association between lameness and the RHpE score. This study highlighted the need to educate owners and trainers about lameness recognition and the RHpE.

Overall, gait and RHpE data from 491 horses have been assessed and documented. These comprised non-lame horses, lame horses, and lame horses before and after diagnostic anaesthesia had been used to abolish pain causing lameness or abnormalities of canter [1,2,3,4,5,6,7,8,9,10,11]. These data provide strong evidence that a RHpE score of ≥8/24 is likely to reflect the presence of musculoskeletal pain, although some lame horses score <8/24. The results of application of the RHpE by 23 assessors have been documented, including skilled assessors [1,2,5,6,8,9], equine veterinarians after preliminary training [4], an equine physiotherapist after on-line training and additional repeatability assessment [7], and non-trained assessors from a variety of equine-related professions [3]. When the RHpE was applied to 20 horses by 10 veterinarians and one expert assessor, there was excellent consistency in overall agreement for total scores among raters (Intra-class correlation coefficient (ICC) 0.97, *p* < 0.001, Confidence Intervals (CI) 0.95, 0.99), and, for exact agreement between individual observers, there was good to moderate agreement (ICC 0.7, *p* < 0.001, CI 0.56, 0.84) [4].

In order to provide even greater validity for use of the RHpE to differentiate horses with and without musculoskeletal pain, assessment of a larger number of pain-free horses is ideally required. This will strengthen our ability to educate owners, trainers, judges, veterinarians and other equine professionals, such as saddle fitters, about the power of the RHpE. Horses competing in elite level dressage, in which gait quality is of paramount importance, should provide such a population of horses.

There is considerable debate about the social license to compete with horses [12,13,14,15]. In addition, there has been much controversy about the head and neck position of dressage horses and the practice of Rollkur (marked flexion of the neck so that the horse’s nose is close to its chest) which has been used in training and warm-up [16,17,18]. Although the Fédération Equestre Internationale (FEI) rules for dressage state repeatedly that the neck should be raised with the poll as the highest point, with the front of the head vertical [19], a trend towards favourable marking of horses with their heads behind a vertical position has been noted. There was a higher frequency of occurrence of the head being behind a vertical position in the 2008 dressage World Cup final compared with the Olympic Games in 1992 [20].

Moreover, the FEI rules of dressage state “agitation of the tail, is mostly a sign of nervousness, tension or resistance on the part of the Horse and must be taken into account by the Judges in their marks for every movement concerned, as well as in the collective mark. The degree of the submission (obedience) is also demonstrated by the way the Horse accepts the bit” [19].

In a pilot study, the RHpE was applied to video recordings of one FEI World Cup Grand Prix dressage qualifying competition (venue A) at which there were 17 competitors (unpublished data). The horses ranged in age from 7 to 18 years (median 13 years). One horse showed transient, mild right forelimb lameness in left half pass. Otherwise, no lameness was detected in the sample of horses. The RHpE scores ranged from 0–6, with a median score of 2. The head was behind the vertical ≥10° for periods of ≥10 s in 10 horses (59%). Repeated unprovoked tail swishing was observed in 3 horses and an additional 10 horses showed repeated tail swishing in synchrony with the application of spur cues. Repeated mouth opening with separation of the teeth for periods of ≥10 s was seen in 8 horses (53%). A repeatability study for data acquisition from these video recordings demonstrated excellent agreement. It was possible to detect relatively subtle signs, such as repeated exposure of the sclera.

It was believed that collection of data from more elite dressage competitions would:

1. Provide additional evidence that the RHpE can be used to differentiate horses with and without musculoskeletal pain when ridden.
2. Provide evidence that at elite level the majority of dressage horses are comfortable in their work.
3. Provide data that may be useful for training of dressage judges and trainers and coaches.
4. Place us in a better position to educate owners, trainers, judges, veterinarians and other equine professionals about the value and power of the RHpE.

The objective of this study was to apply the RHpE to video recordings of horses performing a Grand Prix dressage test at FEI World Cup qualifying competitions and World Cup Finals to determine the range and median RHpE scores. It was hypothesised that this should be a group of horses with a low incidence of musculoskeletal pain; thus, RHpE scores would be consistently <8. Additional aims were to compare RHpE scores with percentage scores awarded by the judges, to determine the frequency of occurrence of each of the abnormal behaviours observed and to compare these and other observations concerning gait with the guidelines for judging FEI dressage.

## 2. Materials and Methods

### 2.1. Data Acquisition

The study was approved by the Ethics Review Panel of the Royal College of Veterinary Surgeons (2020–26). Data were acquired from video recordings of FEI World Cup dressage competitions (*n* = 7) in the Western League from 2018–2020 and the 2018 and 2019 World Cup Finals. The qualifications to compete in the Western League World Cup qualifying competitions are summarised in Supplementary Information 1. Video recordings were available via YouTube and/or FEI-TV. Only video recordings which included all competitors at the competition were used.

The video recordings at each venue had been acquired in a standardised manner, so that each horse at a particular venue and among venues was viewed from similar perspectives for each movement of the FEI Grand Prix dressage test (Supplementary Information 2). The duration of the FEI Grand Prix test is approximately 6.5 min. The video recordings at each competition were assessed in chronological order, in real-time, and the RHpE (Table 1) was applied by a trained assessor (SD). Each recording could be stopped and replayed in real-time. Some horses repeatedly swished their tail throughout the test; others did so sporadically; it was noted, when possible, if tail swishing coincided with the application of a spur cue. Additional free-hand comments were recorded concerning whether particular movements provoked specific behaviours (for example, ears back and an intense stare in piaffe) and how movements were performed relative to the FEI guidelines (for example, regularity of steps in piaffe, front of the head in front of the vertical in extended walk) (Supplementary Information 3) [19].

Each dressage test was judged by a panel of five (seven at the finals) professional judges, situated at different standardised locations around the 60 × 20 m arena. Each movement within the test was allocated a score (0–10; Supplementary Information 4) according to FEI guidelines [19]. Some movements have a coefficient of 2 (for example, half-pass) and are, thus, marked out of 20. In addition, there is a final collective mark for the “rider’s position and seat and correctness and effect of the aids”, also with a coefficient of 2. The final total score out of a potential sum of 460 was the mean score of the five (seven) judges. The judges’ final percentage score was announced at the end of each test and recorded. The placings of all riders were announced at the end of each competition and recorded. Age, breed, and sex data for all horses were available from the FEI web site and were recorded.

All horses had to wear double bridles and a cavesson or crank cavesson noseband. Noseband fit was checked according to FEI guidelines [21]. This was performed by a steward, using an index finger at the side of the nasal bones, at the termination of each test. All riders had to wear spurs, and their appropriateness was also assessed by the steward.

For each venue the RHpE scores were compared with the judges’ final percentage scores. Although the judging panels varied among competitions, each horse’s final score was the mean of the scores of five to seven professional judges who underwent regular training to maintain consistency of marking. It was, therefore, considered reasonable also to compare the RHpE scores with the judges’ final percentage scores. The data for horse RHpE scores from all competitions were assessed individually and pooled. A small proportion of the horses appeared in the data set more than once, permitting the assessment of the consistency of their behaviours.

On a separate occasion the video recordings were re-reviewed to analyse the movement “halt at C-immobility-rein back five steps-proceed immediately at collected trot”, according to the presence or absence of the following 13 features: head behind vertical ≥10° in halt, mouth open with separation of the teeth in halt, halt not square, halt not sustained, halt not at marker, head behind vertical ≥10° in rein back, mouth open in rein back, lack of diagonal steps in rein back, hindlimb(s) dragged in rein back, forelimb(s) dragged in rein back, rein back crooked, incorrect number of steps, other (for example, walked after rein back). The movement “extended walk” was also assessed to determine if the front of the head was behind a vertical position ≥10° for >3 steps.

### 2.2. Data Analysis

Data were imported from Microsoft Excel (Office 365; Microsoft Corporation, Redmond, WA, USA) into statistical software Stata (IC v.13.0; StataCorp LP, College Station, TX, USA) for coding and analyses. Normality of distribution for continuous variables was visually assessed with histograms, including overlaid normal and kernel density plots, and using the formal Shapiro-Wilk test. Continuous variables (age and dressage judges’ score) and ordinal variables (RHpE score and number of errors for the movement “halt-immobility-rein back five steps-collected trot”) were described as medians with IQR and range. Categorical variables were described as proportions (%). The median RHpE and dressage judges’ percentage scores were described for each of the nine venues and for all venues combined. First, the nature of the relationship between RHpE and dressage judges’ scores was visualised using a two-way scatter plot. Due to the RHpE scores being non-normally distributed (Shapiro-Wilk *p* value = 0.036), the Spearman’s rank correlation coefficient was used to test if a correlation existed between the RHpE and the dressage judges’ scores and to estimate the strength of the relationship. The absolute magnitude of the observed correlation coefficient was interpreted as: 0.00 to 0.10 (negligible correlation), 0.10 to 0.30 (weak correlation), 0.40 to 0.69 (moderate correlation), 0.70 to 0.89 (strong correlation) and 0.90 to 1.00 (very strong correlation) [22]. The frequency of occurrence of the RHpE behaviours was described across all nine venues combined to determine the most commonly observed behaviours. The consistency of RHpE scores within horses evaluated competing at more than one venue was assessed by describing their RHpE scores at each venue and how consistently or inconsistently the most frequently observed behaviours were expressed. Observations from the extended walk, piaffe, and passage and canter flying change and pirouette movements were described.

## 3. Results

Data were available for 150 competitors, comprising 123 horses, 18 of which were assessed at two venues, five at three venues, three at four venues and one at five venues. Age, sex, and breed data are summarised in Table 2. The median age for all competitors was 13 years (IQR 12, 15; range 7, 19 years). The majority of competitors were Warmbloods (98%), with geldings predominating (58%). Three competitors were eliminated, one because of forelimb lameness and two because of blood in the mouth. Of 147 competitors which completed the test, transient mild forelimb lameness was seen in 14 (9.5%) in half pass, extended trot or passage; 17 competitors (11.6%) showed unilateral or bilateral hindlimb toe drag in passage or extended trot. Nine competitors (6.1%) exhibited variable temporal and spatial separation of the hindlimbs in sequence flying changes.

### 3.1. The RHpE Scores

The total RHpE scores and their relationship with the judges’ scores for all competitors are summarised in Table 3. The median RHpE score for the nine venues ranged from 2/24 to 3/24, with overall scores ranging from 0–4 to 0–7. There was a significant negative correlation between the RHpE score and the judges’ score at four of the nine venues.

**Figure 1:**
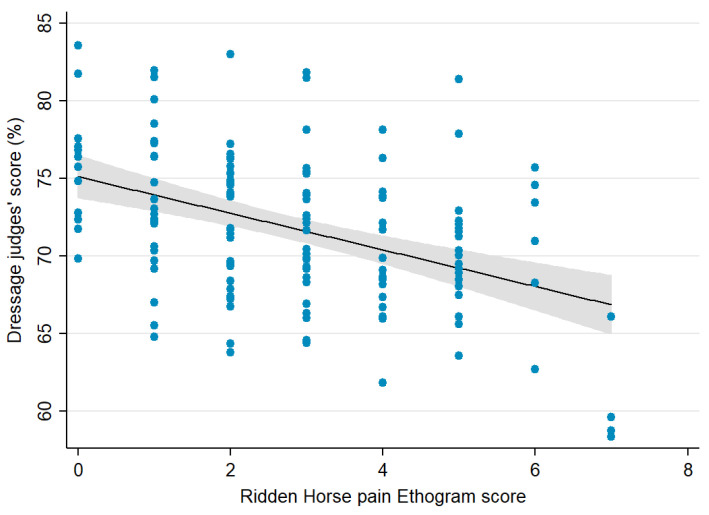
The relationship between the Ridden Horse Pain Ethogram score (0–24) and the dressage judges’ score (percentage) for 147 competitors at World Cup Grand Prix Dressage Western League qualifiers (*n* = 7) and championships (*n* = 2), showing the individual scores, fitted linear prediction and 95% confidence interval (CI). There was a moderate negative correlation (Spearman rho −0.3956, *p* < 0.001). Note the y-axis does not start at zero.

Overall, combining data from all the venues, the median RHpE score was 3 out of 24 (IQR 1, 4; range 0, 7). There was a moderate negative correlation (Spearman rho −0.40, *p* < 0.001) between the RHpE score and the judges’ percentage score (Figure 1).

### 3.2. Frequency of Occurrence of the Behaviours of the RHpE

Mouth open with separation of the teeth for ≥10 s and the front of the head behind the vertical ≥10° for ≥10 s were the most frequent behaviours observed at all venues; an intense stare for ≥5 s (three venues), repeated tail swishing (three venues), ears back for ≥5 s (two venues), and repeated head tilt (one venue) were the other most frequently occurring behaviours. The data for all venues combined are summarised in Table 4.

Overall, repeated tail swishing not in synchrony with cues applied by the rider using spurs was observed in 42 competitors (28.6%). In addition, a further 53 competitors (36.1%) showed repeated tail swishing which appeared to be in synchrony with spur cues.

### 3.3. Consistency of RHpE Scores within Horses Evaluated Competing at More Than One Venue

The total RHpE scores for horses competing at more than one venue are summarised in Table 5. The majority of horses had differences in total scores of ≤2; however, three horses (horses 3, 17 and 23, Table 4) had differences of 4.

However, the consistency of individual behaviours which were observed was variable (Table 6).

### 3.4. The Movement “Halt-Immobility-Rein Back Five Steps-Collected Trot”

The errors in the movement “halt-immobility-rein back-proceed in collected trot” are summarised in Table 7 (video footage of this movement was missing for one horse for this analysis). Although the median number of errors was small, only 2/13, the range was large (0,8). Moreover, several major errors were seen frequently including halt not square (*n* = 52, 35.6%), halt not at marker (*n* = 25, 17.1%), rein back head behind vertical ≥10° (*n* = 92, 63.0%), rein back mouth open with separation of the teeth (*n* = 48, 32.9%), and rein back incorrect number of steps (*n* = 34, 23.3%).

### 3.5. Analysis of Extended Walk

The front of the head was behind a vertical position ≥10° for >3 steps in 53 competitors (36.1%). For nine competitors (6.1%), the head was behind the vertical only in extended walk and not in other gaits or movements.

### 3.6. Piaffe and Passage

The majority of competitors (61.9%, *n* = 91) showed gait abnormalities in passage and/or piaffe. The most frequent modification of passage was the almost simultaneous placement of the hindlimbs to the ground, for example, the left hindlimb, followed by the rapid placement of the right hindlimb caudal to the left hindlimb, so both were bearing weight together.

There were many different ways in which horses modified their gait in piaffe, failing to comply with the definitions of correctness (see Supplementary Information 3). These included an irregular hindlimb rhythm (reduced stance phase of one hindlimb); reduced flexion of the hindlimbs with the feet staying close to the ground, or leaving the toe of a hind foot on the ground (i.e., failure to fully remove the feet from the ground); failing to maintain suspension throughout the period which a hindlimb should be non-weightbearing, with the toe of the foot touching the ground, usually not loading the heel, with a double beat; loading one hindlimb and then rapidly placing the toe of the contralateral hindlimb to the ground followed by the heel (no double beat); placing both hindlimbs to the ground almost simultaneously; being croup high and placing the new weightbearing limb caudal to the contralateral hindlimb which was in the stance phase; placing one hindlimb in front and then behind the contralateral hindlimb; bringing both hindlimbs further under the trunk, with increased slope of the thoracolumbosacral region; bringing the forelimbs back and the hindlimbs forward so that all four limbs were bunched under the trunk; slowing the rhythm; progressive forward movement. Occasionally, rearing or stopping were observed. Errors in the transitions from piaffe to passage included breaking to walk or yanking the head and neck down and forwards.

### 3.7. Canter Flying Changes and Canter Pirouettes

Canter flying changes (two time and/or one time) were incorrect, characterised by being croup high, swinging excessively from side to side, missed changes or repeated close temporal and spatial placement of the hindlimbs in 30 competitors (20.4%). Canter pirouettes were abnormal, usually characterised by close temporal placement of the hindlimbs and often associated with the head being considerably behind a vertical position in 18 competitors (12.2%).

## 4. Discussion

As hypothesised the median RHpE score was low, consistent with the absence of many of the signs associated with musculoskeletal pain in the majority of horses, and a relatively narrow spectrum of behaviours was observed compared with lame horses [1,2,3,4,5,6,7]. The most frequently observed behaviours were the front of the head being behind a vertical position >10° for ≥10 s, mouth opening with separation of the teeth for ≥10 s, an intense stare for ≥5 s and repeated tail swishing, similar to the most common behaviours observed in elite event horses during the warm-up for the dressage phase at 5* three-day events [9].

### 4.1. Head behind a Vertical Position

There has been considerable previous discussion about head and neck position, particularly in relationship with Rollkur, in dressage horses [16,17,18], although the widespread use of draw reins in showjumping horses has largely been ignored (Dyson, S. Personal observations). In the initial development of the RHpE the incidence of the front of the head being behind a vertical position was lower in lame horses compared with non-lame dressage and showjumping horses which had been trained to work with their heads in this position [1]. A skillful rider can alter the position of a non-lame horse’s head from this position to the front of the head being in a vertical position, whilst maintaining contact via the reins with the bit [18,23]. However, it had previously been observed that some horses with musculoskeletal pain adapt by positioning the head behind the vertical and the rider is unable to alter this position [1]. The reins may be loose, or the rider may comment that the rein contact is abnormally light, meaning reduced rein tension. In more difficult manoeuvres, for example, a circle of 10 metres diameter versus 20 metres diameter, the head may go more behind a vertical position in a horse with musculoskeletal pain. However, when musculoskeletal pain is relieved by diagnostic anaesthesia, the front of the head usually becomes more vertical, with the rider reporting a stronger rein tension. For this reason, this behaviour was retained in the final RHpE. In a convenience sample of 60 horses believed by their riders to be working comfortably, the front of the head behind a vertical position had a 7% higher prevalence in lame versus non-lame horses [6].

It was notable in the current study that, in movements which the horses consistently found more difficult (rein back, passage, piaffe, one-time flying changes and canter pirouettes), the frequency of occurrence of head behind a vertical position increased and the angle behind the vertical position increased. In addition, the behaviours ears back ≥5 s and intense stare ≥10 s were only seen during passage and piaffe in some horses. When the top placed 15 horses at the 1992 Olympic Games and the 2008 World Cup Final were compared, there was a significantly greater likelihood of the head being behind a vertical position during piaffe and passage in 2008 than in 1992 [20], which was associated with higher marks, despite the FEI Rules for Dressage stating that “being behind the bit” demonstrates lack of submission” [19]. However, in the current study, some horses had the head behind the vertical position in all gaits, including extended walk. The FEI Rules for Dressage state that for extended walk “The Athlete allows the Horse to stretch out the head and neck (forward and downwards) without losing contact with the mouth and control of the poll. The nose must be clearly in front of the vertical.” [19]. In the current study, the front of the head was behind the vertical >10° for >3 steps of extended walk in 36% of horses. The judges’ marks for each movement were not available, so it is not possible to determine if horses were penalised for this incorrect head and neck posture.

The FEI Rules for Dressage also state that for all movements other than extended walk the front of the head should be in a vertical position or just in front of a vertical position with the poll the highest point of the horse’s neck [19]. However, the dorsal neck muscle/crest development in 15 horses (10 stallions, 4 geldings and 1 mare) in the current study meant that it was physically not possible for the poll to be the highest point of the neck with the front of the head in a vertical position. In these horses, the highest point of the horse’s neck was estimated to be at the third or fourth cervical vertebrae.

### 4.2. Mouth Opening with Separation of the Teeth

There was a high frequency of occurrence of mouth opening with separation of the teeth for ≥10 s. It has previously been shown that mouth opening with separation of the teeth occurs more frequently in lame horses compared with non-lame horses [1,2,6,24], and the frequency decreased after resolution of pain using diagnostic anaesthesia (61% in lame horses, 32% after lameness was abolished using diagnostic anaesthesia) [3]. However, mouth opening with separation of the teeth may also occur in horses without musculoskeletal pain [1,6,9,24]. In the current study this behaviour occurred in 68% of competitors, compared with 44% of non-lame sports horses [6] and 45% of 5* three-day event horses warming-up for dressage [9]. In the latter studies the majority of horses had snaffle bits. The influence of a double bridle on mouth opening behaviour, therefore, merits further investigation.

Mouth opening in ridden horses has been described as conflict behaviour [25], but to what extent it is influenced by the presence or absence of a bit, the type of the bit and its fit, rein tension and rider skill is currently unknown. It was previously documented that there was a greater frequency of mouth opening when horses were ridden compared with being lunged in a bridle, suggesting that it was related to the horse being ridden [24]. There is ongoing debate about bits and their potential to cause pain and/or injury [26]. Many of the behaviours suggested to reflect bit pain [25] were not observed in the current study, and, specifically, there was a low frequency of occurrence of the tongue being out and no evidence of the tongue being over the bit. In Dutch dressage horses, there was a low frequency of occurrence of lesions in the commissures of the mouth [27]. A large majority had double bridles, but there was no difference in oral lesions between those horses ridden in a double bridle (8.3% of 2569 horses) versus a snaffle bridle (9.7% of 291 horses).

Mouth opening and exposure of the tongue outside the oral cavity could potentially be associated with discomfort related to the noseband. However, noseband fit was checked by the FEI Steward at each competition according to FEI guidelines [21], although the methods used do not follow the recommendations of the International Society of Equitation Science, which advise the use of a taper gauge on the dorsal aspect of the nasal bones [28]. Compared with the data from elite three-day event horses [9], in which the rules for dressage allow a much broader spectrum of noseband types, many with greater potential to restrict mouth opening (for example, crank flash, grackle, drop nosebands), the frequency of mouth opening with separation of the teeth was less for the event horses (44%) compared with the dressage horses in the current study (68%), with little difference in the frequency of tongue out (three day event horses 8%, dressage horses 5%). These data suggest that the noseband is not having a major influence on mouth opening or exposure of the tongue.

The FEI Rules for Dressage do not specifically mention mouth opening, but do state that the degree of the submission (which is equated with obedience) is also demonstrated by the way the horse accepts the bit, with light and soft contact and a supple poll [19]. In some horses in the current study, there was rhythmic opening of the mouth in synchrony with the gait. It is speculated that this is a reflection of changing pressures in the oral cavity induced by the bit. Rein tension varies over the stride cycle in trot and is thought to reflect the cyclic movements of the horse’s head and neck relative to the trunk [29,30]. In a study of seven dressage horses assessed on a treadmill in sitting trot in a snaffle bridle, mouth opening was seen substantially more frequently when the horses were ridden ‘on the bit’ compared with unrestrained [31]. When “on the bit”, the mouth movements were associated with the suspension phase of the trot, coinciding with the maximum distance between the rider’s hands and the mouth. However, in the final multivariable analysis, head and neck position did not affect mouth movements, reflecting the complex interactions that occur between the horse and the rider.

### 4.3. Tail Swishing

Repeated tail swishing was seen in a total of 65% of competitors, with or without an association with the application of spur cues. The FEI Guidelines [19] indicate that tail agitation should influence the score for each movement in which the behaviour is observed. Although repeated tail swishing is part of the RHpE, it has also been regarded as a conflict behaviour in ridden horses and has been used as a marker of ”rideability”, or as a response to stress [17,18,23,25,32].

### 4.4. RHpE Scores and Performance

Despite all the horses in the current study being elite athletes, there was a moderate correlation between the RHpE score and the judges’ score. However, there were some notable outliers (Figure 1); the winner at one venue scored 82% and had a RHpE score of 5. Moreover, the median RHpE score for the top ranked five competitors at each venue was 2 (range 0, 6) compared with the overall median score of 3 (range 0, 7). It is notable that these athletes range in age from 8 to 19 years; a larger number of horses would be required to provide a study of adequate power to relate age to the RHpE score. When considering consistency of the RHpE scores within horses assessed on more than one occasion several variable factors need to be taken into account, including the interval between competitions, progression in training, the potential for horses to have received treatments for underlying problems, training intensity prior to the competition, the distance travelled to the competition, the disruption in the horse’s day to day management, and the effect of ageing. The day-to-day variance in RHpE scores in non-lame and lame horses has not yet been assessed in a large number of horses.

### 4.5. Correctness of Movements Compared with the FEI Requirements

There was a high frequency of occurrence of riding or training errors in the movement “Halt-immobility-rein back 5 steps-proceed at collected trot”, including failure to halt at the marker, halt not square, halt not sustained and an incorrect number of rein back steps, which should result in potentially unnecessary loss of marks. The overall incidence of head behind vertical ≥10° (68%) (often ≥30°) and mouth open with separation of the teeth (33%) was disturbingly high. The duration of rein back was <10 s; therefore, these behaviours may not have been included in the RHpE score for those horses which only exhibited these abnormalities during rein back. This is a potentially difficult movement to judge accurately because it progresses quickly, and the video recordings were usually replayed more than once to ensure accurate recording of events.

Passage and piaffe are modifications of trot [33,34,35,36,37,38], and errors were observed in 62% of competitors. Piaffe is a particularly challenging movement to perform correctly [34], and many abnormal variations were observed, with some horses never performing correct steps according to the FEI requirements. The inability to consistently perform correct steps may relate to inability to learn how to sustain static equilibrium and maintain balance, while lifting a diagonal pair of limbs [38]. This may be compounded by lack of musculoskeletal strength and coordination, fatigue, or discomfort, or a combination thereof.

The canter pirouette is a movement with a four-beat gait, with the outside hindlimb, the inside hindlimb, the outside forelimb and the inside forelimb placed in sequence [39]. The most common errors were the close temporal placement of both hindlimbs, with the head low and behind a vertical position and slowing of the rhythm. The close temporal placement of the hindlimbs is presumably a mechanism to reduce the time spent loading a single hindlimb and may also reflect lack of musculoskeletal strength and coordination, fatigue or discomfort, or a combination thereof.

The piaffe, passage, and rein back sequences were the movements most frequently performed the worst. The marking sequence for the four movements passage, transitions passage-piaffe-passage, piaffe (with a coefficient of 2), and passage occurs three times in the test, representing 150 marks. This comprises 33% (150/460) of the possible marks. Despite this, there were horses with clear hindlimb irregularities in passage and piaffe, together with head behind the vertical and mouth opening, which overall scored >70%, equating to “fairly good” (Supplementary Information 4). It would appear that, despite consistency among judges, the judging does not reliably follow the FEI guidelines [19].

However, it must also be borne in mind that the overall performance of horses reflects their training, how they are presented by their riders, neuromuscular coordination, fitness and freedom from discomfort. Given the high frequency of occurrence of the head being behind a vertical position throughout a large proportion of the test it seems likely that this to some extent reflects training, but it does not necessarily indicate the practice of Rollkur. The biomechanical effects of the head positioned behind the vertical on the function of the rest of the body and musculoskeletal health have not been investigated scientifically, but there are persuasive arguments against it based on repeated clinical observations [40,41]. Other factors which may influence both performance and behaviour include the position, weight distribution and balance of the rider, rider strength, the method of application of cues, misinterpretation of cues by the horse, external stressors, and daily training methods.

Although the median RHpE score was low, there was evidence that some horses were showing signs consistent with episodic discomfort. There are many potential reasons including primary musculoskeletal pain, especially associated with biomechanically more demanding movements; pain induced by the tack; alterations in the rider’s weight distribution; and the manner in which cues were applied. The reason(s) in any particular horse could only be determined by careful clinical assessment by a team of suitably qualified professionals. However, this requires that the presence of a potential problem is recognised by the team producing the horse and that appropriate advice is sought. This approach may result in both improved performance and enhanced equine welfare.

### 4.6. Limitations

The study had some limitations. The identity of the competitors could not be blinded. Lighting prohibited the assessment of the eye expression in some horses. Excessive white “froth” around the lips, possibly induced by feeding sugar-lumps prior to the test, prevented assessment of mouth opening with separation of the teeth in a small proportion of horses. There was limited footage from directly in front or behind; therefore, the assessment of both straightness and tail carriage was restricted. It was not possible to obscure obvious errors, for example, failure to perform a flying change. The video recordings were assessed by only one experienced observer; however, a high level of repeatability of application of the RHpE by a trained assessor has previously been demonstrated [1,4]. Gait assessment was performed by the same assessor, so there is potential for bias; however, the judges’ scores were unknown, and the statistical analyses were performed completely independently.

## 5. Conclusions

This study provided evidence that, at elite level, most of the Grand Prix dressage horses studied were comfortable in the majority of their work, based on the RHpE scores. However, some horses showed episodic discomfort, and identification of the cause may enhance equine welfare and performance. There was a moderate negative correlation between the RHpE and judges’ scores. The study also provided information about movements that were repeatedly not performed according to FEI guidelines, with descriptions of the errors, which may be useful for the training of dressage officials, judges, riders, trainers and coaches. It highlighted the frequency of occurrence of horses working with the front of the head ≥10° behind a vertical position for ≥10 s and mouth opening with separation of the teeth for ≥10 s. The influence of a double bridle compared with a snaffle bridle merits further investigation.

## Figures and Tables

**Table 1:** Summary of the 24 behaviours of the Ridden Horse Pain Ethogram (adapted from Dyson et al. [1]).

Definitions of Each of the Behaviours
1. Repeated changes of head position (up/down), not in rhythm with the trot
2. Head tilted or tilting repeatedly
3. Head in front of vertical (≥30°) for ≥10 s
4. Head behind vertical (≥10°) for ≥10 s
5. Head position changes regularly, tossed or twisted from side to side, corrected constantly
6. Ears rotated back behind vertical (both or one only) for ≥5 s; repeatedly lay flat
7. Eye lids closed or half closed for 2–5 s; frequent blinking
8. Sclera exposed repeatedly
9. Intense stare (glazed expression, “zoned out”) for ≥5 s
10. Mouth opening ± shutting repeatedly with separation of teeth, for ≥10 s
11. Tongue exposed, protruding or hanging out, and/or moving in and out repeatedly
12. Bit pulled through the mouth on one side (left or right), repeatedly
13. Tail clamped tightly to middle or held to one side
14. Tail swishing large movements: repeatedly up and down/side to side/circular; repeatedly during transitions
15. A rushed gait (frequency of trot steps >40/15 s); irregular rhythm in trot or canter; repeated changes of speed in trot or canter
16. Gait too slow (frequency of trot steps <35/15 s); passage-like trot
17. Hindlimbs do not follow tracks of forelimbs but repeatedly deviated to left or right; on 3 tracks in trot or canter
18. Canter repeated leg changes change of leg in front and/or behind; repeated strike off wrong leg; disunited
19. Spontaneous changes of gait (e.g., breaks from canter to trot or trot to canter)
20. Stumbles or trips more than once; repeated bilateral hindlimb toe drag
21. Sudden change of direction, against rider direction; spooking
22. Reluctance to move forwards (has to be kicked ± verbal encouragement), stops spontaneously
23. Rearing (both forelimbs off the ground)
24. Bucking or kicking backwards (one or both hindlimbs)

**Table 2:** Summary of age in years (median, interquartile range (IQR) and range), sex, and breed data for 150 competitors competing at nine venues at World Cup Grand Prix dressage Western European qualifying competitions (*n* = 7) and two World Cup Grand Prix dressage finals, 2018–2020. The data for 27 horses which competed at least two or more venues are included.

Venue	Number	Median Age	Age IQR	Age Range	Sex			Breed	
					Gelding	Stallion	Mare	Warmblood	Other
1	14	12	12, 15	9, 17	7	5	2	14	0
2	18	14	13, 15	10, 18	11	5	2	18	0
3	18	13	12, 15	11, 17	14	3	1	18	0
4	16	12.5	12, 14	9, 16	10	4	2	15	Lusitano
5	16	10.5	10, 12.5	9, 18	12	2	2	15	Lusitano
6	19	14	11, 15	9, 17	11	7	1	19	0
7	18	14.5	12, 17	10, 17	7	9	2	18	0
8	14	13.5	12, 16	10, 19	8	4	2	13	Friesian
9	17	13	12, 15	8, 18	7	7	3	17	0
Total	150	13	12, 15	8, 19	58.0%	30.7%	11.3%	97.8%	2.2%

**Table 3:** The relationship between the judges’ final total percentage score and the Ridden Horse Pain Ethogram (RHpE) score (0–24) assessed using Spearman’s correlation for horses (*n* = 147) competing at nine venues at World Cup Grand Prix dressage Western European qualifying competitions (*n* = 7; venues 1, 3–6, 8, 9) and two World Cup Grand Prix dressage finals (venues 2 and 7), 2018–2020. Significant negative correlations are highlighted in bold.

Venue	Median RHpE Score	RHpE Score IQR*	RHpE Score Range	Median of Judges’ Total Scores %	IQR * of Judges’ Total Scores %	Range of Judges’ Total Scores %	Spearman’s Rho	*p* Value
1	2.5	2, 5	0, 6	74.2	69.5, 76.2	62.7, 81.8	−0.80	0.001
2	3	2, 4	0, 7	72.0	69.2, 74.7	58.3, 81.4	−0.33	0.17
3	3	2, 4	0, 7	69.6	67.4, 73.4	59.6, 81.5	0.02	0.95
4	3	1, 5	0, 5	71.6	69.1, 73.7	63.6, 75.3	−0.55	0.03
5	2.5	1, 4.5	0, 7	70.5	66.1, 73.4	61.8, 81.6	−0.42	0.11
6	3	2, 4	0, 5	72.1	69.3, 74.7	65.6, 83.0	−0.57	0.017
7	2	1, 4	0, 4	74.6	69.9, 77.0	66.0, 81.8	−0.34	0.16
8	2	1, 3	0, 7	68.9	67.2, 74.8	58.7, 77.6	−0.59	0.026
9	2	1, 5	0, 6	74.6	71.8, 76.6	64.4, 83.6	−0.26	0.31

* IQR = interquartile range.

**Table 4:** Summary of the frequency of occurrence of each of the behaviours (*n* = 24) of the Ridden Horse Pain Ethogram for all venues combined, for competitors (*n* = 147) at World Cup Grand Prix Dressage Western League qualifying competitions (*n* =7) and finals (*n* = 2).

Behaviour	Number	Percentage
Mouth open with separation of the teeth for ≥10 s	100	68.0
Front of head behind vertical ≥10° for ≥10 s	98	66.7
Intense stare ≥5 s	44	29.9
Repeated tail swishing not in synchrony with spur aids	42	28.6
Ears back behind vertical ≥5 s	36	24.5
Repeated head tilt	30	20.4
Spontaneous change of gait	13	8.8
Repeated stumbling or bilateral hindlimb toe drag	10	6.8
Repeated exposure of the sclera	10	6.8
Repeated exposure of the tongue	7	4.8
Head moved from side to side	3	2.0
Spontaneous change of direction; spooking	3	2.0
Head movement up and down, not in synchrony with the trot rhythm	2	1.4
Bucking	1	0.7
Rearing	1	0.7
Reluctance to go forwards	1	0.7
Crooked, on 3 tracks	1	0.7
Crooked tail, held to one side	1	0.7
Eyes partially closed 2–5 s; repeated blinking	1	0.7
Gait too slow	0	0
Rushed gait	0	0
Repeated incorrect strike off in canter	0	0
Bit pulled through to one side	0	0
Front of head in front of vertical ≥30° ≥10 s	0	0

**Table 5:** Summary of the total Ridden Horse Pain Ethogram scores for 27 horses which competed at more than one World Cup Grand Prix Dressage Western League qualifying competition (*n* = 7) or final (*n* = 2).

Horse Number	Number of Venues	RHpE Scores (0–24)
1	5	0, 0, 0, 2, 3
2	4	1, 1, 2, 2
3	4	0, 2, 2, 4
4	4	1, 2, 2, 3
5	3	2, 3, 5
6	3	1, 1, 2
7	3	5, 5, 6
8	3	1, 3, 4
9	3	2, 4, 4
10	2	2, 3
11	2	1, 0
12	2	2, 5
13	2	4, 5
14	2	4, 5
15	2	1, 1
16	2	5, 6
17	2	2, 6
18	2	5, 5
19	2	2, 2
20	2	2, 2
21	2	0, 1
22	2	4, 4
23	2	1, 5
24	2	0, 1
25	2	2, 5
26	2	3, 6
27	2	4, 5

**Table 6:** Consistency of observations of the 24 behaviours of the Ridden Horse Pain Ethogram displayed by 27 horses assessed on two (*n* = 18), three (*n* = 5), four (*n* = 3), and five (*n* = 1) occasions competing at World Grand Prix Dressage Western League qualifying competitions (*n* = 7) or finals (*n* = 2). The six most frequently observed behaviours (observed in >10 horses) are in bold.

Behaviour	Horses Showing Behaviour at Least Once	Horses Showing Inconsistency	Horses Showing Consistency
**Mouth opening ± shutting repeatedly with separation of teeth, for** **≥10 s**	**24**	**10**	**14**
**Head behind vertical (≥10 degrees) for** **≥10 s**	**21**	**7**	**14**
**Intense stare (glazed expression, “zoned out”) for** **≥5 s**	**15**	**10**	**5**
**Tail swishing large movements: repeatedly up and down / side to side / circular; repeatedly during transitions**	**12**	**10**	**2**
**Head tilted or tilting repeatedly**	**11**	**9**	**2**
**Ears rotated back behind vertical (both or one only) for** **≥5 s; repeatedly lay flat**	**11**	**8**	**3**
Stumbles or trips more than once; repeated bilateral hindlimb toe drag	6	5	1
Sclera exposed repeatedly	5	4	1
Spontaneous changes of gait (e.g., breaks from canter to trot or trot to canter)	3	3	0
Repeated changes of head position (up/down), not in rhythm with the trot	2	2	0
Rearing (both forelimbs off the ground)	2	2	0
Tongue exposed, protruding or hanging out, and/or moving in and out repeatedly	1	1	0
Tail clamped tightly to middle or held to one side	1	1	0
Canter repeated leg changes change of leg in front and/or behind; repeated strike off wrong leg; disunited	1	1	0
Sudden change of direction, against rider direction; spooking	1	1	0
Head in front of vertical (≥30 degrees) for ≥10 s	0	0	0
Head position changes regularly, tossed or twisted from side to side, corrected constantly	0	0	0
Eye lids closed or half closed for 2–5 s; frequent blinking	0	0	0
Bit pulled through the mouth on one side (left or right), repeatedly	0	0	0
A rushed gait (frequency of trot steps >40/15 s); irregular rhythm in trot or canter; repeated changes of speed in trot or canter	0	0	0
Gait too slow (frequency of trot steps <35/15 s); passage-like trot	0	0	0
Hindlimbs do not follow tracks of forelimbs but repeatedly deviated to left or right; on 3 tracks in trot or canter	0	0	0
Reluctance to move forwards (has to be kicked ± verbal encouragement), stops spontaneously	0	0	0
Bucking or kicking backwards (one or both hindlimbs)	0	0	0

**Table 7:** Summary of the frequency of occurrence of errors in the movement “halt at C-immobility-rein back five steps-proceed in collected trot” for all venues combined (146 competitors) for World Cup Grand Prix Western League qualifying competitions (*n* = 7) and finals (*n* = 2). The median score was 2/13 (interquartile range 1, 3; range 0, 8).

Error	Number	Percentage
Halt head behind vertical	26	17.8
Halt mouth open with separation of the teeth	13	8.9
Halt not square	52	35.6
Halt not sustained	14	9.6
Halt not at marker	25	17.1
Rein back head behind vertical ≥10°	92	63.0
Rein back mouth open with separation of the teeth	48	32.9
Rein back lack of diagonal steps	18	12.3
Rein back, hindlimb(s) dragged	6	4.1
Rein back, forelimb(s) dragged	13	8.9
Rein back crooked	8	5.5
Rein back incorrect number of steps	34	23.3
Other *	10	6.7

* Other = stepped back with one hindlimb after halting; raised head before or during rein back; variable rhythm of steps in rein back; walked between rein back and collected trot; jumped into collected trot; head in front of vertical in transition to collected trot; mouth open with separation of teeth in collected trot.

## Data Availability

Data are available from the authors on reasonable request.

## Supplementary Materials

The following are available online at https://www.mdpi.com/article/10.3390/ani11051187/s1, Supplementary information 1: Fédération Equestre Internationale qualifications for competing in Western European World Cup Grand Prix qualifying competitions, Supplementary information 2: The Fédération Equestre Internationale Grand Prix Dressage test, Supplementary information 3: A summary of the key points related to specific gaits and movements performed in the Grand Prix dressage test according to the Fédération Equestre Internationale Rules for Dressage and guidelines for their assessment, Supplementary information 4. The criteria for judging as documented in the Fédération Equestre Internationale Dressage Rules, 25th edition, effective 1st January 2014; including updates effective 1st January 2019.
