# Supplementary material for: Application of the Ridden Horse Pain Ethogram to Elite Dressage Horses Competing in World Cup Grand Prix Competitions
Source: Animals (Basel). 2021 Apr 21;11(5):1187. doi: 10.3390/ani11051187 (PMC8143096; doi:10.3390/ani11051187)
Supplement: Supplementary file 1 [file animals-11-01187-s001.zip › animals-1183295-supplementary.pdf]

Article

Application of the Ridden Horse Pain Ethogram to Elite Dressage Horses Competing in World Cup Grand Prix Competitions

Sue Dyson [1] and Danica Pollard [2]

[1] The Cottage, Church Road, Market Weston, IP22 2NX Diss, UK; sue.dyson@aol.com

[2] The Rodhams, Rodham Road, Christchurch, PE14 9NU Wisbech, UK; drdee.pollard@gmail.com

* Correspondence: sue.dyson@aol.com; Tel.: +44 (0)7860 826028

Citation: Dyson, S.; Pollard, D. Application of the Ridden Horse Pain Ethogram to Elite Dressage Horses Competing in World Cup Grand Prix Competitions. *Animals* **2021**, *11*, x. https://doi.org/10.3390/xxxxx

Academic Editor: Sue M. McDonnell

Received: 30 March 2021
Accepted: 19 April 2021
Published: date

Publisher's Note: MDPI stays neutral with regard to jurisdictional claims in published maps and institutional affiliations.

Copyright: © 2021 by the authors. Submitted for possible open access publication under the terms and conditions of the Creative Commons Attribution (CC BY) license (http://creativecommons.org/licenses/by/4.0/).

Supplementary material:

Supplementary information 1

Fédération Equestre Internationale qualifications for competing in Western European World Cup Grand Prix qualifying competitions

Art. 5—WESTERN EUROPEAN LEAGUE

1. In Western Europe events may be organised at a limited number of two (2) CDI-Ws per National Federation (NF). The FEI Dressage World Cup™ qualifying events must be exclusively indoor events.

2. Invitations

•The title defender has the automatic right to compete in every qualifier.

•The Top 10 Western European League athletes of the FEI World Ranking List (the "WR list"). If one of the athletes decides not to compete at an event, that athlete will be replaced by the next placed on the WR list, provided the replacement athlete is ranked within the top 30 of the WR list.

•For the events organized in the first half of the season (e.g., events starting before 31 December) the WR list from September will be used and for the second half of the season (e.g., events starting since 1st January) the WR list from December will be used. (max 1 horse per athlete).

•The Organising Committee (OC) has the right to limit the number of starters per foreign NF to three (3) or four (4) per NF. This must be mentioned in the approved schedule.

•The home NF has the right to nominate up to five (5) athletes unless this quota has been filled as per above.

•One FEI wildcard for an athlete of a NF not already entered.

•The OC has the right to invite other athletes until the maximum number of athletes invited in the schedule has been filled.

Supplementary information 2

The Fédération Equestre Internationale Grand Prix Dressage test.

	Markers At Which Movement Performed	Instructions	Score	Coefficient	Directions To Judges
1	A<br>X<br>X<br>C	Enter in collected canter<br>Halt - immobility - salute<br>Proceed in collected trot<br>Collected trot	10		Quality of paces, halt, and transitions. Straightness. Contact and poll.
2	C<br>HXF<br>FAK	Track to the left<br>Extended trot<br>Collected trot	10		Regularity, elasticity, balance, of hindquarters, overtrack. Lengthening of frame. Both transitions.
3	KB	Half-pass to the righ	10	2	Regularity and quality of trot, uniform bend, collection, balance, fluency, crossing of legs.
4	BH<br>HC	Half-pass to the left<br>Collected trot	10	2	Regularity and quality of trot, uniform bend, collection, balance, fluency, crossing of legs.
5	C	Halt - immobility<br>Rein back 5 steps an[illegible] immediately procee[illegible] in collected trot	10		Quality of halt and transitions. Throughness, fluency, straightness. Accuracy in number of diagonal steps.
6	MV	Extended trot	10		Regularity, elasticity, balance, energy of hindquarters, overtrack. Lengthening of frame. Transition to extended trot.
7	VKD	Passage	10		Regularity, cadence, collection, self-carriage, balance, activity, elasticity of back and steps. Transition to passage.
8	D	Piaffe 12 to 15 steps	10	2	Regularity, taking weight, self-carriage, activity, elasticity of back and steps. Specific number of diagonal steps.
9	D	Transitions passage - piaffe - passage	10		Maintenance of rhythm, collection, self-carriage, balance, fluency, straightness. Precise execution.
10	DFP	Passage	10		Regularity, cadence, collection, self-carriage, balance, activity, elasticity of back and steps.

11	PH	Extended walk	10	2	Regularity, suppleness of back, activity, overtrack, freedom of shoulder, stretching to the bit. Transition into walk.
12	HCM	Collected walk	10	2	Regularity, suppleness of back, activity, shortening and heightening of steps, self-carriage.
13	M	Proceed in passage Transition collected walk - passage	10		Fluency, promptness, self-carriage, balance, straightness.
14	MRI	Passage	10		Regularity, cadence, collection, self-carriage, balance, activity, elasticity of back and steps.
15	I	Piaffe 12 to 15 steps	10	2	Regularity, taking weight, self-carriage, activity, elasticity of back and steps. Specific number of diagonal steps.
16	I	Transitions passage - piaffe - passage	10		Maintenance of rhythm, collection, self-carriage, balance, fluency, straightness. Precise execution.
17	ISE	Passage	10		Regularity, cadence, collection, self-carriage, balance, activity, elasticity of back and steps.
18	E<br>EKAF	Proceed in collected canter left<br>Collected canter	10		Precise execution and fluency of transition. Quality of canter.
19	FXH<br>HCM	On the diagonal 9 flying changes of leg every 2nd stride<br>Collected canter	10		Correctness, balance, fluency, uphill tendency, straightness. Quality of canter before and after.
20	MXK	Extended canter	10		Quality of canter, impulsion, lengthening of strides and frame. Balance, uphill tendency, straightness.
21	K<br>KA	Collected canter and flying change of leg<br>Collected canter	10		Quality of flying change on diagonal. Precise, smooth execution of transition.
22	A<br>Between D & G<br>G<br>C	Down the centre line<br>5 half-passes to either side of centre line with flying change of leg at each change of direction, the first half-pass to the left and the last to the left of 3 strides, the others of 6 strides<br>Flying change of leg<br>Track to the right	10	2	Quality of canter. Uniform bend, collection, balance, fluency from side to side. Symmetrical execution. Quality of flying changes.

23	MXK<br>KA	On the diagonal 15 flying changes of leg every stride<br>Collected canter	10	2	Correctness, balance, fluency, uphill tendency, straightness.<br>Quality of canter before and after.
24	A<br>L	Down the centre line<br>Pirouette to the left	10	2	Collection, self-carriage, balance, size, flexion and bend. Correct number of strides (6-8). Quality of canter before and after.
25	X	Flying change of leg	10		Correctness, balance, fluency, uphill tendency, straightness.<br>Quality of canter before and after.
26	I<br>C<br>CM	Pirouette to the right<br>Track to the right<br>Collected canter	10	2	Collection, self-carriage, balance, size, flexion and bend. Correct number of strides (6-8). Quality of canter before and after.
27	M<br>MR	Collected trot<br>Collected trot	10		Fluency; precise, smooth execution of transition. Collection.
28	RK<br>K<br>KA	Extended trot<br>Collected trot<br>Collected trot	10		Regularity, elasticity, balance, energy of hindquarters, overtrack.<br>Lengthening of frame.<br>Both transitions. Collection.
29	A<br>DX	Down the centre line<br>Passage	10		Regularity, cadence, collection, self-carriage, balance, activity, elasticity of back and steps. Transition to passage.
30	X	Piaffe 12 to 15 steps	10	2	Regularity, taking weight, self-carriage, activity, elasticity of back and steps.<br>Specific number of diagonal steps.
31	X	Transitions passage - piaffe - passage	10		Maintenance of rhythm, collection, self-carriage, balance, fluency, straightness.<br>Precise execution.
32	XG	Passage	10		Regularity, cadence, collection, self-carriage, balance, activity, elasticity of back and steps.
33	G	Halt - immobility - salute	10		Quality of halt and transition. Straightness.<br>Contact and poll.

Supplementary information 3

A summary of the key points related to specific gaits and movements performed in the Grand Prix dressage test according to the Féderation Equestre Internationale Rules for Dressage and guidelines for their assessment.

The halt: The neck should be raised with the poll as the highest point and the nose-line slightly in front of the vertical. While remaining "on the bit" and maintaining a light and soft contact with the athlete's hand, the horse may quietly chew the bit and should be ready to move off at the slightest indication of the athlete.

Extended walk: The horse covers as much ground as possible, without haste and without losing the regularity of the steps. The hind feet touch the ground clearly in front of the hoof prints of the fore feet. The athlete allows the horse to stretch out the head and neck (forward and downwards) without losing contact with the mouth and control of the poll. The nose must be clearly in front of the vertical.

Rein back: During the entire exercise, the horse should remain "on the bit", maintaining its desire to move forward. Anticipation or precipitation of the movement, resistance to or evasion of the contact, deviation of the hindquarters from the straight line, spreading or inactive hind legs, and dragging forefeet are serious faults.

The Pirouette: (half-pirouette) is a turn of three hundred and sixty (360) degrees (hundred and eighty—180—degrees) executed on two tracks, with a radius equal to the length of the horse and the forehand moving around the haunches.

Pirouettes (half-pirouettes) are usually carried out at collected walk or canter but can also be executed at piaffe.

At the pirouette (half-pirouette) the forefeet and the outside hind foot move around the inside hind foot. The inside hind leg describes a circle as small as possible.

At whatever pace the pirouette (half-pirouette) is executed, the horse, slightly bent in the direction in which it is turning, remains "on the bit" with light contact, turning smoothly around, and maintaining sequence and timing of footfalls of that pace. The poll remains the highest point during the entire movement.

During the pirouettes (half-pirouettes), the horse should maintain its activity (walk also included) and never move backwards or sideways.

In executing the pirouette or the half-pirouette in canter, the athlete should maintain lightness of the horse while accentuating the collection. The horse's hindquarters are well engaged and lowered and show a good flexion of the joints. An integral part of the movement is the quality of the canter strides before and after the pirouette. The strides should show an increased activity and collection before the pirouette and the balance should be maintained at the end of the pirouette.

Aims of the pirouette and half-pirouette in canter: to demonstrate the willingness of the horse to turn around the inside hind leg on a small radius, slightly bent in the direction of the turn while maintaining the activity and the clarity of the canter, the straightness and the balance before and after the figure and clear canter strides during the turn. In the pirouette or half-pirouette in canter, the Judges should be able to recognize a real canter stride although the feet of the diagonal – inside hind leg, outside front leg – are not touching the ground simultaneously.

Passage is a measured, very collected, elevated and cadenced trot. It is characterised by a pronounced engagement of the hindquarters, a more accentuated flexion of the knees and hocks, and the graceful elasticity of the movement. Each diagonal pair of legs is raised and returned to the ground alternately, with cadence and a prolonged suspension.

In principle, the height of the toe of the raised forefoot should be level with the middle of the cannon bone of the other supporting foreleg. The toe of the raised hind foot should be slightly above the fetlock joint of the other supporting hind leg.

The neck should be raised and gracefully arched with the poll as the highest point and the nose line close to the vertical. The horse should remain light, and soft "on the bit" without altering the cadence. The impulsion remains lively and pronounced.

Irregular steps with the hind or front legs, swinging the forehand or the hindquarters from one side to the other, as well as jerky movements of the forelegs or the hind legs, dragging the hind legs or double beat in the moment of suspension are serious faults.

Piaffe is a highly collected, cadenced, elevated diagonal movement giving the impression of remaining in place. The horse's back is supple and elastic. The hindquarters are lowered; the haunches with active hocks are well engaged, giving great freedom, lightness and mobility to the shoulders and forehand. Each diagonal pair of legs is raised and returned to the ground alternately, with spring and an even cadence.

In principle, the height of the toe of the raised forefoot should be level with the middle of the cannon bone of the other supporting foreleg. The toe of the raised hind foot should reach just above the fetlock joint of the other supporting hind leg.

The neck should be raised and gracefully arched, with the poll as the highest point. The horse should remain "on the bit" with a supple poll, maintaining soft contact. The body of the horse should move in a supple, cadenced and harmonious movement.

Piaffe must always be animated by a lively impulsion and characterised by perfect balance. While giving the impression of remaining in place, there may be a visible inclination to advance, this being displayed by the horse's eager acceptance to move forward as soon as it is asked.

Moving even slightly backwards, irregular or jerky steps with the hind or front legs, no clear diagonal steps, crossing either the fore or hind legs, or swinging either the forehand or the hindquarters from one side to the other, getting wide behind or in front, moving too much forward or double-beat rhythm are all serious faults.

Submission does not mean subordination, but an obedience revealing its presence by a constant attention, willingness and confidence in the whole behaviour of the horse, as well as by the harmony, lightness, and ease it is displaying in the execution of the different movements.

The degree of the submission is also demonstrated by the way the horse accepts the bit, with light and soft contact and a supple poll. Resistance to or evasion of the athlete's hand, being either "above the bit" or "behind the bit" demonstrate lack of submission. The main contact with the horse's mouth must be through the snaffle bit.

Putting out the tongue, keeping it above the bit or drawing it up altogether, as well as grinding the teeth or agitation of the tail, are mostly signs of nervousness, tension or resistance on the part of the horse and must be taken into account by the Judges in their marks for every movement concerned, as well as in the collective mark.

The position of the head and neck of a horse at the collected paces is naturally dependent on the stage of training and, to some degree, on its conformation. It is distinguished by the neck being raised without restraint, forming a harmonious curve from the withers to the poll, which is the highest point, with the nose slightly in front of the vertical. At the moment the athlete applies his aids to obtain a momentary and passing collecting effect, the head may become more or less vertical. The arch of the neck is directly related to the degree of collection.

Supplementary information 4

The criteria for judging as documented in the Fédération Equestre Internationale Dressage Rules, 25th edition, effective 1st January 2014; including updates effective 1st January 2019.

All movements, and certain transitions from one to another, which have to be marked by the Judges, are numbered on the Judge's sheet.

Each movement is marked from zero (0) to ten (10) by each Judge, zero (0) being the lowest and ten (10) the highest mark.

10 excellent; 9 very good; 8 good; 7 fairly good; 6 satisfactory; 5 sufficient; 4 insufficient; 3 fairly bad; 2 bad; 1 very bad; 0 not performed

All half marks from 0.5–9.5 may also be used both for movements and the collective mark, at the discretion of the judge.

'Not executed' means that practically nothing of the required movement has been performed.

Collective mark: After the athlete has finished his performance, a collective mark is awarded for the athlete's position and seat; correctness and effect of the aids (General impression). The collective mark is awarded from zero (0) to ten (10).
